# The Public Ledger Almanac for 1892

**Published:** 1892-03

**Authors:** 


THE PUBLIC LEDGER ALMANAC FOR 1892. Geo. W. Childs, Publisher. Chestnut and Sixth Streets, Philadelphia.

This is the twenty-third annual issue of this well known publication, which has become, as well stated in the preface, "a home-book of reference and a treasury of useful information on local and general subjects and events." It is furnished free of cost.

